# Growing Up in Poverty, Growing Old in Infirmity: The Long Arm of Childhood Conditions in Great Britain

**DOI:** 10.1371/journal.pone.0144722

**Published:** 2015-12-16

**Authors:** Gindo Tampubolon

**Affiliations:** Cathie Marsh Institute for Social Research, University of Manchester, Manchester, United Kingdom; TNO, NETHERLANDS

## Abstract

**Background:**

The ageing population poses a tremendous challenge in understanding the sources of inequalities in health. Though they appear to be far removed, childhood conditions are known to be inextricably linked with adult health, and in turn on health in later life. The long arm of childhood conditions hypothesis is often tested using recollection of childhood circumstances, but such subjective recall can yield potentially inaccurate or possibly biased inferences. We tested the long arm hypothesis on three outcomes in later life, arrayed from objective to subjective health, namely: gait speed, episodic memory and mental health.

**Methods and Findings:**

We used the English Longitudinal Study of Ageing 2006 enriched with retrospective life history (*N* = 5,913). To deal with recall problems two solutions, covariate measurement and endogenous treatment models, were applied. Retrospective childhood material lack includes growing up without running hot or cold water, fixed bath, indoor lavatory and central heating. Adjustment is made for an extensive set of confounders including sex, age, adult health, wealth, education, occupation, social support, social connections, chronic conditions, smoking, drinking, and physical exercise. It is found that material poverty when growing up shows no association with health when growing old, assuming accurate recall. Once recall problems are controlled, we found that childhood material poverty changes inversely with later life health.

**Conclusion:**

A poorer childhood goes with slower gait, poorer memory and more depression in later life. This result provides a further impetus to eliminate child poverty.

## Introduction

In 2008 some 2500 Britons aged 50 were asked to recall the number of rooms and people in the house when they were 11, in order to assess overcrowding. Only one in three got both numbers right (compared to their mothers’ original answers when visited those years ago; both numbers are required to measure overcrowding). It is also found that using the retrospective data the odds of suffering from asthma at age 50 decreases with overcrowding, while using the objective data the odds increases [1]. Apparently asthmatic middle-aged people report a more spacious childhood.

If middle-aged people are prone to recall error and recall bias about their childhood material conditions, what about older people (50 to 89 years old)? And if the retrospective conditions are related problematically with respiratory health, how do the conditions relate to other health outcomes of objective and subjective kinds, such as gait speed, cognitive function and mental health? These are not idle questions. In fact they are at the heart of the hypothesis of the long arm of childhood conditions.

The hypothesis maintains that a lack of resources during childhood leads to lower educational and occupational attainment as well as lower health and well-being later in the life course, particularly in adulthood [2]. Childhood material lack is hypothesised to associate with health outcomes long into adult life [3].

There are a number of ways in which childhood conditions can leave a lasting impact [4, 5]. The pathway model posits that low socioeconomic status during childhood leads to lower initial socioeconomic positions in early adulthood. In turn, these lower positions lead to lower adult health status. Essentially, because socioeconomic positions in early adulthood (including education and occupation) summarise a range of conditions, no direct association between childhood status and adult health remains. In another way, the life course model posits both direct and indirect effects of childhood condition on adult health. Disadvantaged socioeconomic conditions during childhood may be contemporaneously harmful to health, and this health disadvantage may persist. Thus childhood conditions lead to both restricted life chances (lower socioeconomic status) as well as poorer health in adulthood.

The deleterious effect of childhood hardship, even during childhood itself, has also been examined. Some authors [6] noted that financial strains, through pain they inflict on parents, can lead to increased expression of hostility, reduced warmth and impaired skilful child rearing.

It must be noted here that the space allocated to these negative links between childhood hardship and adult health does not amount to a law of nature. Social scientists have also demonstrated that some children can thrive even after exposure to hardship and deprivation [7].

Nevertheless, the predominant theme is of the harmful effect of childhood hardship on adult health [8]. One study, notable for its design, has been conducted on a sample of nearly 700 African-American twins [9]. When using twins sample, individual effects or genetic predispositions are controlled for much more satisfactorily than when using a sample from the general population. The authors identified childhood and adulthood financial hardships as contributors to cumulative disadvantage. Childhood and adulthood financial hardships together are more harmful to adult health than either experience alone.

The links in these path models and life course models have been found not only in developed countries, but also in developing countries using both prospective and retrospective study design [7, 10]. Almost all these studies have been unable to dismiss the notion that the effects of childhood conditions reach long into adulthood.

The long arm hypothesis has an extensive reach not only across space but, some believe, also across the life course, to touch outcomes well into later life. The evidence thus far is equivocal. In 2000, for example, Finns aged 56–66 suffering early life stress sustained from being evacuated to Sweden and Denmark during World War II, compared to those who stayed in the country, showed higher systolic and diastolic blood pressure [11]. Meanwhile in Sweden researchers found no association between childhood conditions and later life health outcomes (self-report of general health, mobility problems, cardiovascular diseases and musculoskeletal disorders) once educational attainment earlier in the life course was considered [12].

There is then an urgent need to understand how childhood conditions are linked to health in later life, not only at adulthood. With the population across the world visibly ageing, there is increasing interest in measuring the long arm of childhood conditions.

Keen to enable empirical investigation of this hypothesis, prospective longitudinal ageing studies with their collection of objective and contemporaneous information are increasingly enriched with retrospective data about childhood circumstances. This combination of prospective and retrospective data should make it possible to ask first order questions, i.e. whether childhood conditions actually relate to later life health and well-being, and through what mechanisms. The main alternatives to these enriched-ageing studies, namely cohort studies such as the National Child Development Studies 1958 or the Medical Research Council cohort 1946, are simply not yet long enough, or not sufficiently widely available for testing the long arm hypothesis.

Such mixed prospective-retrospective data come with potential recall error and recall bias [1]. Recall error manifests in responses being randomly inaccurate, while recall bias manifests in responses being tainted by current health levels, such as when asthmatic older people report rosier childhood. Progress in testing the long arm hypothesis is seriously hampered if a study takes recall information as entirely accurate and makes no provision for the potential recall problems. In response to this, the literature has demonstrated the use of the classical covariate measurement model to deal with inaccurate recall, and the endogenous treatment model to deal with recall bias [13, 14].

This article reports application of both improved techniques when testing the long arm of childhood conditions hypothesis in Britain, particularly affecting gait speed, episodic memory, and mental health of people aged 50–99. There have been few empirical tests of the hypothesis using nationally representative samples. So this gap invites the attempt to understand whether in Britain such enduring associations are at work.

The paper aims first to examine the long term associations between childhood material lack and a range of health outcomes that cover the objective-subjective spectrum (inclusive). Second, it aims to show the consequences of ignoring recall problems when estimating the associations, by comparing results from standard models and those from the improved techniques. Most importantly, it aims to weigh the contribution of growing up in material poverty to the variations in older people’s physical, cognitive and mental health.

In achieving these aims this paper contributes to the literature on ageing and on life course in four ways. To the bias first found using the 1958 cohort at age 50 [1], it extends the age range in the British population when recollection of childhood material conditions is demonstrably tainted by health levels. Then, exploiting the range of health outcomes from objective to subjective ones, it characterises the recall problems, enabling a tailored approach when examining different kinds of outcomes (objective or subjective) in later life. Third, some biases are shown to be removed successfully by applying endogeneous treatment model, giving results more consistent with our view of the mechanisms of human development, and thus enriching our understanding of it. Lastly, it uses an extensive set of covariates, paving a way to estimating the net association with childhood material conditions some half century in the past.

To delineate the scope of our investigation four questions will be raised. What are the associations between material lack at age 10 with health outcomes of gait speed, episodic memory and mental health in later life, assuming accurate recall? How much is childhood poverty associated with physical or mental infirmity at old age, once provision for recall problems is made? How will they change according to the objective-subjective distinction of health outcomes under study? What are the theoretical and methodological implications for life course and ageing studies?

We next introduce the English Longitudinal Ageing Study and briefly describe improved techniques to dealing with the problematic retrospective information typical in life course and ageing studies. Health outcomes in later life are chosen following Fisher’s suggestion (in [15] pp. 5–6) to be elaborate when testing a hypothesis, hence the three outcomes together span the objective to subjective spectrum. We shall then compare results from the standard model without correction and those from the improved techniques for each health outcome.

The discussion commences with general rather than specific patterns. Afterwards, since the three health outcomes are chosen from the objective to subjective spectrum, the results will be lined up, yielding lessons to apply when dealing with retrospective data in diverse settings.

## Materials and Analysis

### The English Longitudinal Study of Ageing—Life history data

ELSA is an ongoing longitudinal study of ageing in England, starting in 2002 and conducted biennially since. It began with respondents of the Health Survey for England in 1998, 1999 and 2001. The sample is representative of community-dwelling older people and this has been maintained through replenishment [16–18]. As the main ageing study in England, it is closely comparable with its sister study, the US Health and Retirement Survey. The study covers individual and household characteristics, physical, cognitive and mental health, social connection and social support, wealth, and health behaviours. Every four years a nurse visit complements the standard survey and results in extra biomedical information.

This study used the third wave, which uniquely provided information on childhood circumstances, enabling us to construct the main exposure. This information includes whether the following material facilities were not available: running hot water, running cold water, fixed bath, central heating and indoor lavatory. After summing up, the main exposure ranges from 0 (lacking none) to 5 (lacking all material facilities). Other information on childhood circumstances is used as indicators or covariates in the two techniques; this information includes (a) number of books in the home at age 10, (b) numbers of rooms and people in the home at age 10 (both pieces are used to tag overcrowding or more people than rooms [19]), (c) whether or not financial hardship has been experienced in the past and at what age (both pieces are used to indicate whether financial hardship was experienced at age 10).

Apart from the main exposure (childhood material lack) and its indicators or covariates, we chose three health outcomes with a view towards testing the long arm hypothesis elaborately. The outcomes are deliberately arrayed along the objective—subjective spectrum: gait speed, episodic memory, and depression as a measure of mental condition. All entered as continuous variables, gait speed was measured by a nurse (m/sec), episodic memory is the sum of ten words recalled immediately and after a few minutes delay (ranges 0–20), and depression is collected using CES-D instrument (ranges 0–8). To ease understanding all three outcomes are coded in one direction: the higher the better. So we converted the CES-D score as follows: CES-D = 8 to mental health score = 0 or the most depressed; CES-D = 7 to mental health score = 1, and so on. Uniformly, therefore, the higher the scores (of gait speed, episodic memory, mental health), the healthier the individual.

In estimating each association between the main exposure and the outcome, an extensive set of covariates are controlled, a set larger than most in the literature [1, 11, 12]. Demographic characteristics include age, age square, sex (Female); socioeconomic status includes wealth tertiles (the poorest as reference), social class (routine manual class and the rest as reference; National Statistics Socio-Economic Classification [20], education (age at leaving school), and marital status (comprising indicators for married/cohabiting, separated, widowed, and single as reference). Social connections are constructed from responses about meeting friends, relatives and children (or speaking, or writing to them). Each of the responses ranges from 0 for never to 6 for three or more times a week; the sum gives a social connections measure ranging from 0 to 18. Social support is constructed as a binary indicator, using responses to whether, when facing serious problems, there is support from their spouses, children, relatives and friends some or a lot of the time; household size is also included to allow examination of net associations of social connections and social support.

Health conditions are captured using a set of indicators including whether the respondent has any cardiovascular problems comprising angina, hypertension, myocardial, congestive heart failure, heart murmur, arrhythmia, diabetes, stroke; has any chronic obstructive pulmonary diseases (COPD) including bronchitis or asthma; and/or any forms of cancer or malignant tumours. Depressive mood may influence cognition or episodic memory in this sample [21], therefore CESD score as a measure of depressive mood is included in explaining episodic memory.

Health behaviours included are smoking (or not) and physical exercise, comprising vigorous, moderate, and mild or less as reference. And for sensitivity analysis we included the number of illness episodes in adulthood as a measure of adult health status.

To avoid potential collinearity, we checked for pairwise correlations and found that nearly all pairs showed weak correlations. The small number of exceptions is reasonable including pairs of wealth categories which are largely and negatively correlated (<-0.40), age—household size (-0.42), and categories of marital status (married/in partnership—divorced, -0.48; married/in partnership—widowed, -0.67), and married/in partnership—household size, 0.44. We try to show the net influence of each of these covariates by including them all, and thus to isolate the net influence of childhood material lack.

Those with complete information are included in the analysis (*N* = 5193). The complete and left-out samples are tested for difference using *t* test (continuous covariates) and *χ* ^2^ test (nominal covariate). Compared to those left out (*N* = 2377) the analytic sample is older (67 versus 57 year, *p* < 0.001), has similar proportions of women (55% versus 56%, *p* = 0.265), is on average wealthier (£71,618 versus £59,352, *p* = 0.005) and has similar levels of self-rated health, i.e. on a range of 1 as poor to 3 as good or excellent (2.7 versus 2.6, *p* = 0.801).

#### Ethics review

The University of Manchester’s institutional review board has exempted this study since it used publicly available anonymised secondary data for research.

### Statistical analysis

In order to avoid the sort of conflicting results referred to in the Introduction, where subjective recall yields probably inaccurate and possibly biased information, the analyses conduct not only the standard linear model but also error-corrected and bias-corrected models. Error or inaccurate recall is corrected using the covariate measurement model, and bias is corrected using the endogenous treatment model [13]. Later, as part of sensitivity analysis, adult health status is included, since adulthood lies midway between childhood and later life. The purpose is to see whether childhood conditions estimates remain robust.

In the case of recall bias (see Eqs 1 and 2 below), respondents are believed to remember their childhood conditions with less than perfect lucidity. However it is suggested this recall is biased or systematically tainted according to the later life health outcome being examined. For instance, the more asthmatic middle-aged people report a roomier childhood home. There may be joint determination of current health and of recalled childhood conditions. The childhood material lack (of facilities) is a function of the other childhood information including the number of books in the house, whether lived in an overcrowded house, and whether financial hardship was experienced during childhood [13].

In the case of recall error (see Eqs 3 and 4 below), respondents are believed to recall their childhood conditions decades in the past, again with less than perfect clarity. It is also conceded that the imperfection is not biased in any direction, as there is no suggestion that, for instance, those with slower gait remember their childhood better. Their recall therefore contains much useful information, though not entirely to be taken at face value. Together, the childhood information including the (lack of) material facilities, the number of books in the house, whether lived in an overcrowded house, and whether financial hardship was experienced during childhood, gives a clearer measure of childhood conditions. Putting them as indicators of a factor allows one to derive a latent factor of childhood conditions, then to use this in estimating its association with later life health [13].

Specifically, the endogeneous treatment model as a technique to deal with recall bias has two parts: health model (Eq 1) and childhood material lack (Eq 2) ([13]:434–436):

$$g(H_{j}|\eta_{j},X_{j})=X_{j}^{\prime }\beta_{1}+\eta_{j}\alpha +1\zeta_{j},$$ (1)

$$f(\eta_{j}|\zeta_{j},Z_{j},X_{j})=X_{j}^{\prime }\beta_{2}+Z_{j}^{\prime }\gamma +\lambda \zeta_{j}.$$ (2)

Both are generalised linear models, and have an additional covariate that is the latent bias, *ζ*_*j*_. Comparable to covariate measurement error below, for identification one of the coefficient is set to one. In this instance, this setting is chosen for the health equation.

The covariate measurement model as a technique to deal with the recall error problem also has two parts: health model (Eq 3) and measurement model (Eq 4) ([13]:418).

$$g(H_{j}|\eta_{j},X_{j})=X_{j}^{\prime }\beta_{1}+\eta_{j}\alpha ,$$ (3)

$$y_{ij}=\eta_{j}+\epsilon_{ij},\;\epsilon_{ij}∼N\left(0,\theta \right),$$ (4)

where *η* is latent childhood material lack and *α* is its effect on health *H* and the *i* indexed indicators of childhood material circumstances. As is standard in factor analytic models (of which the covariate measurement model is one), identification is secured by setting one of the loadings or the variances in the measurement model to one. In this instance, the former is chosen.

To ascertain the potential problems with relying on subjective recall and to demonstrate the improved techniques, three models will be estimated for each health outcome. Thus for gait speed a linear model is estimated, where the count of material lack is the main exposure and the results are given on the left pane (labelled uncorrected) of a three-pane table. The middle pane (labelled bias-corrected) contains estimates from endogenous treatment model while the right pane (labelled error-corrected) displays results using the covariate measurement model. Similarly for episodic memory the results are given in a three-pane table: uncorrected model, bias-corrected with endogenous treatment model, error-corrected with covariate measurement model; likewise with the last outcome or mental health. Additionally, to obtain focus a handful of these estimates are graphed for each model by each outcome. Lastly, because the outcomes vary in their distribution, the coefficient estimates of the main exposure (childhood conditions) are not readily comparable across health outcomes. To aid comparison the estimates were scaled to the outcome’s standard deviation as the denominator. So standardised, only the main exposure estimates are displayed together in the last figure to aid discussion.

These methods to solve recall bias and recall error were run in Latent GOLD 5.0 [22] to explain how much childhood material lack contributes to slower gait speed, poorer episodic memory, and poorer mental health. Significance is set at 5%, Pearson’s correlation is used to asses bivariate associations and root mean squared error (RMSE) is used to compare models.

## Results

The analytic sample (Table 1) consists of 55% females with an average age of 67, more than a third (36%) of whom had routine manual occupations. On average two out of five material facilities were lacking (childhood lack = 2.02), only a couple of books were available (2.38), but only a minority reported experiencing financial hardship (1%), and one in five lived in an overcrowded house (19%).

**Table 1:** Summary statistics of the sample from ELSA 2006 and Life history data (*N* = 5193).

Variable	Mean/Percentage	SD	Min	Max
Gait speed	0.85	0.27	0.1	2
Episodic memory	10.49	3.45	0.0	20
Mental health	6.65	1.83	0.0	8
Sex, Female	55%			
Age	67.10	9.18	53.0	99
Wealth bottom tertile	26%			
Wealth mid tertile	35%			
Wealth top tertile	39%			
Age left school	16.2	2.47	0	29
Routine manual	36%			
Single	5%			
Married/cohabiting	68%			
Separated/divorced	10%			
Widowed	18%			
Social support	99%			
Social connection	10.21	3.71	0.0	18
Household size	2.20	0.96	1.0	10
Cardiovascular disease	13%			
Chronic obstructive pulmonary disease	15%			
Cancer	6%			
Smoking	12%			
Alcohol daily	34%			
Physical exercise, vigorous	19%			
Physical exercise, moderate	64%			
Adult health (illness episodes in adulthood)	0.60	1.01	0	5
Childhood lack facilities	2.02	1.47	0.0	5
Childhood num. books	2.38	1.21	1.0	5
Childhood experience financial hardship	1%			
Chidlhood overcrowding	19%			

Source: The English Longitudinal Study of Ageing 2006.

Bivariate associations in Table 2 show that slower gait speed, lower levels of cognitive function and lower scores on mental health are found among those lacking material facilities during childhood. Though small, the correlations were all statistically significant. If the case of asthmatic middle-aged Britons in the Introduction is instructive, then there is a need to apply multivariate models capable of accounting for various confounders and sensitive to the potential recall problems for each health outcome.

**Table 2:** Bivariate associations (*ρ*) between childhood conditions and health in later life.

	Gait speed	Episodic memory	Mental health
	(*ρ*; *p*)	(*ρ*; *p*)	(*ρ*; *p*)
Childhood lack	−0.122;< 0.001	−0.156;< 0.001	−0.036; 0.009

### Physical health outcome: gait speed

**Fig 1:**
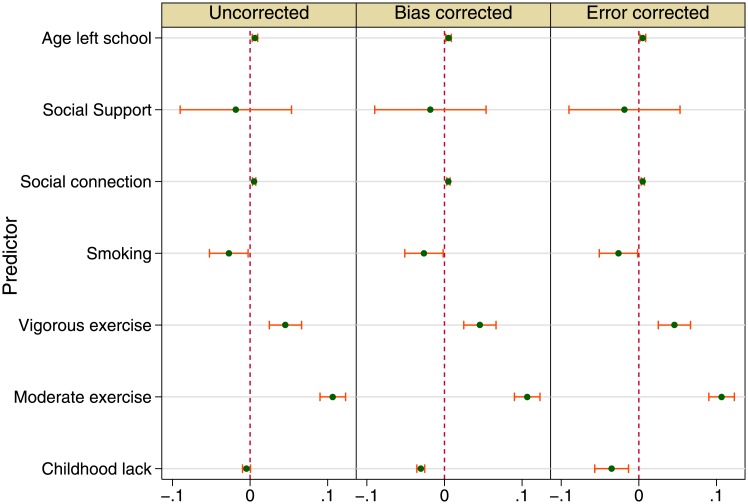
Childhood conditions and health in later life: gait speed (fully adjusted models). Source: The English Longitudinal Study of Ageing 2006.

For concision, Fig 1 shows only a handful of estimates and 95% confidence intervals from fully adjusted models; the three fully adjusted models’ estimates are given in Tables A, B, C in S1 File. The left pane gives net associations of gait speed with age at leaving school, social support, social connection, smoking, physical exercise, and childhood material conditions. The second model estimates seen on the middle pane show what happens when endogeneous treatment technique is applied, while the third model estimates shown on the right pane give estimates after recall error was corrected. In all three models consistent estimates for all risk factors (except for childhood material conditions) are shown, including the finding that higher gait speed goes with exercise of moderate and vigorous levels, compared to no exercise; and that gait speed inversely associates with being a smoker.

When recall problems are ignored, the negative association between childhood conditions and gait speed is merely due to chance (left pane). But when recall problems are recognised and solved using either the endogenous treatment model (middle pane) or measurement error model (right pane), childhood material condition inversely associates with gait speed. The two point estimates are quite similar (−0.031 and −0.035, *p* < 0.001 for both).

Since the bias corrected model (Table A in S1 File) gave the best fit (no other model in the table had smaller RMSE), its coefficients are picked. (This same model cannot be bettered in explaining other health outcomes below.) Significant social determinants of variation in older people’s physical health include education, occupation, wealth and social connection. More education in the form of higher age at leaving school is associated with faster gait speed (0.005, *p* < 0.001). The routine manual workers have slower gait than those of the other classes (−0.039, *p* < 0.001). Wealth gradient in gait speed is also found. Though social support makes no difference to gait speed, more social connection (a structural form of social capital [23–25]) is associated with an increase in gait speed (0.005, *p* < 0.001). Health behaviours which have often been examined in this ageing study show significant results. Being a smoker is associated with slower gait, while drinking more and exercising more are associated with speedier gait.

### Episodic memory

**Fig 2:**
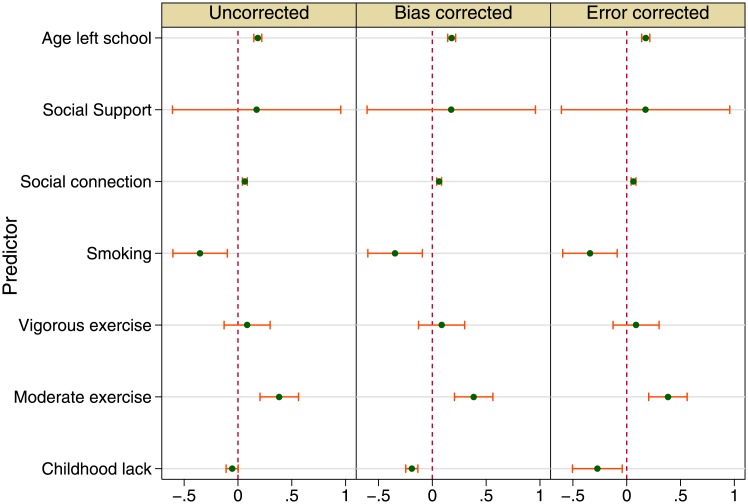
Childhood conditions and health in later life: Episodic memory (fully adjusted models). Source: The English Longitudinal Study of Ageing 2006.

Analogous to the display for gait speed above, the left pane of Fig 2 displays estimates ignoring recall problems, the middle one displays estimates corrected for bias, and the right pane displays estimates corrected for error. Again consistent estimates are seen for all factors in all three panels, giving some ease to the reading of childhood condition estimates. For instance, smoking is associated with reduced memory function, and moderate or vigorous physical exercise confers advantage to memory function, though the latter is not statistically significant.

Like the reading of childhood condition estimates in explaining gait speed above, here we see that ignoring recall problems gives no association between growing up lacking facilities and levels of episodic memory when growing old. But once provision for recall problems is made, both improved techniques give significant and negative estimates: poor childhood conditions go with poorer episodic memory. The coefficients (−0.191 and −0.273, both *p* < 0.05) are different and so are the standard errors, though neither coefficient covers zero (Table B in S1 File).

According to the bias corrected model (Table B in S1 File), social determinants of episodic memory in later life include levels of education and social connections. Staying at school longer is associated with higher levels of episodic memory much later in life (0.180, *p* < 0.001). Social connection or structural social capital is positively associated with scores in the episodic memory test (0.063, *p* < 0.001). Again social support is not significantly associated with episodic memory.

### Subjective health outcome: mental health

**Fig 3:**
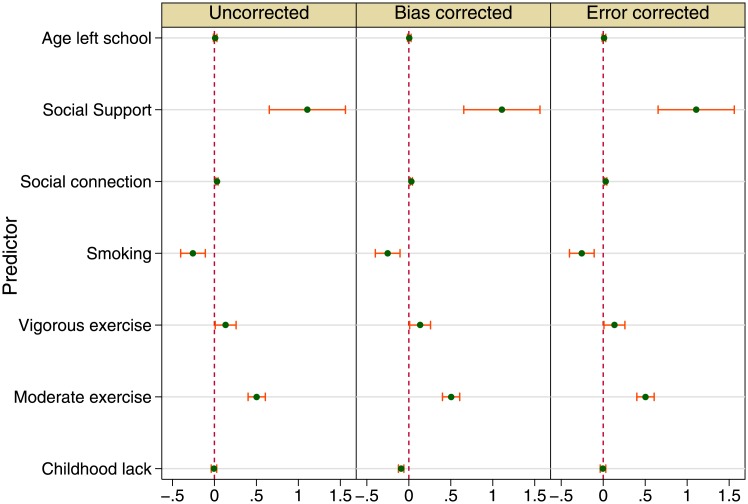
Childhood conditions and health in later life: Mental health (fully adjusted models). Source: The English Longitudinal Study of Ageing 2006.

As regards mental health (Fig 3 and Table C in S1 File), results that confirm expectation and confound it can be seen across the three models. Social connection remains important and associates with better mental health. Unlike the results for gait speed and episodic memory, however, social support is significant here: more social support is reported by people with higher mental health scores (bias corrected coefficient 1.107, *p* < 0.001). Social capital or social connection is again significant in its association with mental health (0.030, *p* < 0.001).

Health risk behaviours of smoking, drinking and physical exercise are significantly associated with mental health; being a smoker is likely to go with a lower mental health score, whereas physical exercise at moderate and vigorous levels vary alongside mental health score.

Like the cases of physical and cognitive functions, the risk factors in Fig 3 show quite stable estimates across the three panes. But it is the main exposure estimates that draw our interest. In explaining mental health, the childhood conditions estimates show no significant difference from zero in both uncorrected and error-corrected models (two outer panes). Without bias correction, the mentally healthy and the depressed report no difference in the levels of material disadvantage during childhood. Once bias is corrected, however, it is found that material disadvantage during childhood is associated with worse mental health in later life (−0.091, *p* < 0.001). This negative association accords with the results about physical and cognitive functions above.

#### Sensitivity analysis

Indirect effects of childhood conditions can be examined by additional adjustment for adult health (specifically adult illness episodes), where such adjustment can reduce the remaining associations with childhood conditions, if indeed adult health is the main channel linking childhood and later life. In the Tables D, E and F in S1 File) we found estimates which display no marked changes to the estimates mentioned above.

## Discussion

Childhood conditions have been implicated in social inequalities in adult health [3] but these are now believed to extend to later life. Conventional investigations of the long arm of childhood conditions which uncritically used subjective recall are suspect, as they may have missed the association between childhood material lack and physical or mental health in later life. Corrections applied here, including the covariate measurement or endogenous treatment models, are able to recover the deleterious associations arising from materially disadvantaged childhood. The whole spectrum of health from physical to mental health (gait speed, episodic memory, and mental health) is affected by childhood conditions long in the past, even after controlling for an extensive set of confounders. The consistency of the harmful associations across this wide range of health outcomes gives confidence in the findings.

What can be learned by examining an array of health outcomes? To gain more insight beyond this consistency we focus exclusively on the childhood conditions estimates across the three health outcomes using all three models. Because the scales of the outcomes differed widely we have rescaled the childhood conditions estimates, using the standard deviation of each outcome as the denominator. This gives the multiple of standard deviation of outcome as the unit of interpretation.

**Fig 4:**
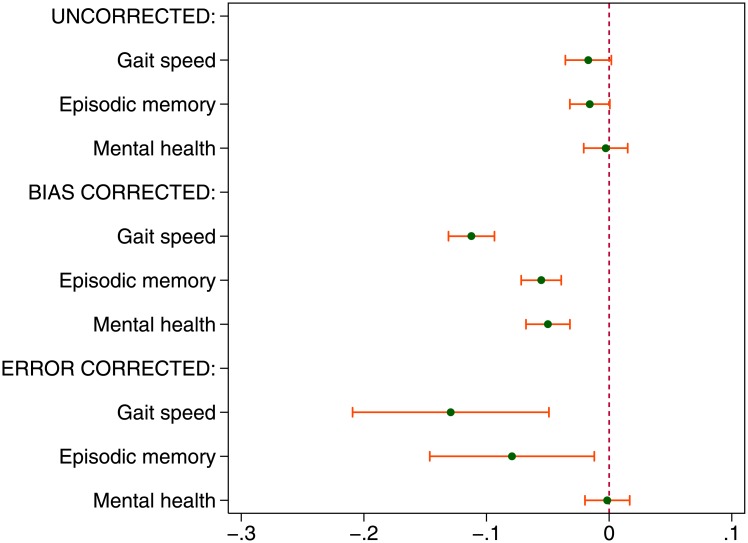
Childhood conditions (standardised estimates) and health in later life: gait speed, episodic memory and mental health (fully adjusted models). Source: The English Longitudinal Study of Ageing 2006.

Above, we have coded all outcomes to point in the same direction so that a higher score is uniformly better for all outcomes; higher gait speeds mark those with better physical health, higher episodic memory scores mark those with better cognitive health, higher mental health scores mark those with better mental health. This also means a negative estimate can be interpreted uniformly as a reduction in health, whatever the outcome under discussion (see Fig 4).

In Fig 4 (top three estimates), no obvious relation appears between childhood conditions and gait speed or episodic memory or mental health, since they are all no different from zero. With error correction (bottom cluster), a reduction by one-seventh of standard deviation in gait speed while growing old is related with growing up with one fewer material facility. And a reduction of nearly one-tenth of standard deviation in episodic memory is associated with growing up without one facility. The bias corrected estimates give sharper inferences (middle cluster), but otherwise the magnitudes given by both improved techniques are comparable except for mental health (more on this below).

This figure is an arrangement motivated by Fisher’s suggestion to elaborate our hypothesis, i.e. to go beyond testing whether childhood condition is associated with later life health. It suggests that childhood condition is associated with later life health from the spectrum of subjective to objective health: mental health—episodic memory—gait speed. It also suggests that the estimates should be aligned to form a recognisable pattern instead of forming a haphazard zig-zag.

Arranged in this way we see that childhood material lack is associated most strongly with physical function (gait speed), followed by episodic memory, and least by mental health, given exactly the same childhood conditions.

What other insights are recovered by applying the two techniques to recall problems? By now it is reasonable to suspect that on balance, subjective recall of childhood material circumstances in ELSA is problematic; either error or bias correction is needed. Given this supposition, for mental health, the error corrected model still failed to give an estimate that was different from that of the uncorrected model (mental health estimates in top and bottom clusters). But the bias correction technique gives a different estimate. Missing one facility when growing up is associated with one-twentieth standard deviation reduction in mental health. The fact that using the endogenous treatment technique for both gait speed and episodic memory also gives a negative association suggests that childhood material lack is credibly and negatively associated with poorer mental health in later life. Thus while objective health outcomes (gait speed and episodic memory) are amenable to either technique, not so with subjective one. It yields only to correction using endogenous treatment technique, since systematic bias or tinting is at work.

The inconsistent results on mental health (bias-corrected versus the other two models) may seem puzzling. To wit, if recall is taken at face value or taken as randomly in error then no difference in childhood material levels is found between the mentally healthy and the depressed. But if provision for bias is made, the materially disadvantaged child is found to have lower mental health in later life. How to reconcile this inconsistency? It is found above that the subjective recall is tinted. This rose-tint varies with mental health so as to render among the depressed a childhood that is materially no different from those of the mentally healthy. As Brown [1] found asthmatic middle-aged people report a roomier childhood, we found depressed older people report a rosier one.

### Mechanisms

The association between childhood material lack and old age infirmities (where the missing facilities include indoor lavatory, running hot and cold water, fixed bath, and central heating) impinges on both biological and social mechanisms that are intertwined. First, children growing up in poor housing conditions are susceptible to infections and various health hazards which put them at a relative disadvantage against their better off peers [26]. These injuries and hazards may be embodied in their physical constitutions or their organs, affecting cognition, stature and lung function. A number of studies reported that lead in the child’s development environment (primarily the home and its environs) is inversely associated with intelligence quotient [27, 28]. The authors [27] also insisted that it is the home environment that has more bearing on the child’s cognitive ability.

### Limitations

This study has some limitations, not least due to its cross-sectional design. Although tempting, the results remain as associations rather than causal effects of childhood material deprivation on later life health and well-being. Later these should be compared to results using child cohort studies like the National Child Development Study 1958, whose members are now in their late 50s. Moreover the focus on material conditions during childhood should not be seen as identifying the material conditions as the only driver or even the main driver that triggers the long arm of childhood conditions. Psychosocial mechanisms have been raised above; though these cannot be investigated directly due to lack of adequate data.

The sufficient information that is able to capture latent material condition during childhood is rarely examined, and the indicators used here for these adjustments are admittedly convenient. These models are thus proposed as a small step in the right direction or viable alternative models, compared to assuming these indicators to be unproblematic or error-free. We also note a possible limitation of these improved techniques in the form of wider standard errors; this may transpire in other settings. Suitable data collection and broader investigation of these aspects should be a priority for future research.

Future research may also consider addressing another limitation of this study, arising from the fact that the sample is made up of those who were alive or survived when ELSA commenced. Those left out from ELSA and the analytic sample may be systematically different in ways that affect the associations between childhood conditions and later life health.

These results nonetheless give us a handle on the direction of the difference. Given the consistent findings that poor childhood conditions are linked with poorer subjective and objective health in later life, and given that those who survived to old age (compared to those who did not) are likely to be healthier or wealthier, then the findings may be seen as conservative estimates of the deleterious associations. The magnitude of the associations could be larger if those who did not survive had had the chance to participate in ELSA. All is not lost however. Mention is made above of the cohort studies coming of ‘old age’. Their childhood conditions and later life health outcomes can be examined for associations together with their chances of surviving to older age in a joint model of health trajectories and attrition. Such a joint model has been applied to ELSA [29]; this is then an entirely sensible direction for future research.

This report is also limited to one country. We are currently examining these associations in 20 European countries using the Survey of Health, Ageing and Retirement in Europe (www.share-project.org).

In conclusion, health in later life cannot escape the childhood conditions [30]; and subjective recall about childhood is not innocuous. But examination of the long arm of childhood conditions can be secured if the inaccuracies of older people’s recall are considered. Once recall problems are taken into consideration, the results found here raise a number of practical, theoretical and political implications. In practice, subjective recall information is useful and covariate measurement and endogenous treatment techniques work well with it. More indicators of childhood conditions, however, can offer much help to these techniques. The results above also suggest that both the covariate measurement and endogenous treatment models are suitable for investigating other objective later life health outcomes, such as lung function measured using spirometry [31], whereas more subjective later life outcomes such as well-being [29, 32] may benefit more from the endogenous treatment technique.

Social epidemiology theory [33, 34] can now add the long arm hypothesis to explain the variation of health outcomes among the different groups of older people. Given the ageing population, where the proportion of people age 65 or over is set to increase, a deeper understanding of population health can only be reached if their childhood conditions are also considered.

The link between childhood conditions and later life demonstrated here provides another impetus to policy aimed at eliminating child poverty. Since life expectancy is likely to increase and the proportion of older people follows suit, it is even more crucial, therefore, to ensure that the childhood stage is not marred by poverty, because growing up in poverty is linked with growing old in infirmity.

## Supporting Information

S1 File Supplementary tables. Three models on gait speed in later life (fully adjusted) (**Table A**). Three models on episodic memory in later life (fully adjusted) (**Table B**). Three models on mental health in later life (fully adjusted) (**Table C**). Three models on gait speed in later life, with adult health (fully adjusted) (**Table D**). Three models on episodic memory in later life, with adult health (fully adjusted) (**Table E**). Three models on mental health in later life, with adult health (fully adjusted) (**Table F**).
(PDF)

S1 Text Attrition and a few other methodological problems in the study of cognitive ageing. (DOCX)
