# Supplementary material for: Growing Up in Poverty, Growing Old in Infirmity: The Long Arm of Childhood Conditions in Great Britain
Source: PLoS One. 2015 Dec 16;10(12):e0144722. doi: 10.1371/journal.pone.0144722 (PMC4682716; doi:10.1371/journal.pone.0144722)
Supplement: S1 File — Three models on gait speed in later life (fully adjusted) (Table A). Three models on episodic memory in later life (fully adjusted) (Table B). Three models on mental health in later life (fully adjusted) (Table C). Three models on gait speed in later life, with adult health (fully adjusted) (Table D). Three models on episodic memory in later life, with adult health (fully adjusted) (Table E). Three models on mental health in later life, with adult health (fully adjusted) (Table F). (PDF) [file pone.0144722.s001.pdf]

S1 File. Supplementary tables

Table A. Three models on gait speed in later life (fully adjusted).

Predictors	Uncorrected	Bias-corrected	Error-corrected
Constant	$0.716 \pm 0.264^{\dagger}$	$0.803 \pm 0.264^{\dagger}$	$0.745 \pm 0.264^{\dagger}$
Sex, Female	$-0.048 \pm 0.008^{\dagger}$	$-0.049 \pm 0.008^{\dagger}$	$-0.050 \pm 0.008^{\dagger}$
Age	0.006 ± 0.007	0.006 ± 0.007	0.006 ± 0.007
Age^2	$0.000 \pm 0.000^{*}$	$0.000 \pm 0.000^{*}$	$0.000 \pm 0.000^{*}$
Household size	0.007 ± 0.006	0.007 ± 0.006	0.007 ± 0.006
Routine manual	$-0.041 \pm 0.009^{\dagger}$	$-0.039 \pm 0.009^{\dagger}$	$-0.038 \pm 0.009^{\dagger}$
Wealth mid-tertile	$0.043 \pm 0.01^{\dagger}$	$0.041 \pm 0.010^{\dagger}$	$0.041 \pm 0.010^{\dagger}$
Wealth top-tertile	$0.088 \pm 0.011^{\dagger}$	$0.086 \pm 0.011^{\dagger}$	$0.086 \pm 0.011^{\dagger}$
Age left education	$0.006 \pm 0.002^{\dagger}$	$0.005 \pm 0.002^{\dagger}$	$0.005 \pm 0.002^{\dagger}$
Married-cohab.	0.038 ± 0.020	0.038 ± 0.020	0.038 ± 0.020
Separated-div.	0.038 ± 0.023	0.037 ± 0.023	0.038 ± 0.023
Widow	0.034 ± 0.021	0.034 ± 0.021	0.034 ± 0.021
Social support	-0.018 ± 0.037	-0.018 ± 0.037	-0.019 ± 0.037
Social connection	$0.005 \pm 0.001^{\dagger}$	$0.005 \pm 0.001^{\dagger}$	$0.005 \pm 0.001^{\dagger}$
CVD	$-0.024 \pm 0.010^{*}$	$-0.024 \pm 0.010^{*}$	$-0.024 \pm 0.010^{*}$
COPD	$-0.039 \pm 0.011^{\dagger}$	$-0.039 \pm 0.011^{\dagger}$	$-0.039 \pm 0.011^{\dagger}$
Cancer	-0.006 ± 0.015	-0.007 ± 0.015	-0.007 ± 0.015
Smoking	$-0.027 \pm 0.013^{*}$	$-0.026 \pm 0.013^{*}$	$-0.026 \pm 0.013^{*}$
Drinking	$0.020 \pm 0.009^{*}$	$0.020 \pm 0.009^{*}$	$0.019 \pm 0.009^{*}$
Exercise, vigorous	$0.046 \pm 0.011^{\dagger}$	$0.046 \pm 0.011^{\dagger}$	$0.046 \pm 0.011^{\dagger}$
Exercise, moderate	$0.107 \pm 0.008^{\dagger}$	$0.107 \pm 0.008^{\dagger}$	$0.107 \pm 0.008^{\dagger}$
Childhood lack facility	-0.005 ± 0.003	$-0.031 \pm 0.003^{\dagger}$	$-0.035 \pm 0.011^{\dagger}$
$RMSE$	0.0038	0.0025	0.0037

Source: The English Longitudinal Study of Ageing.

* $p < 0.05$.

† $p < 0.01$.

Table B. Three models on episodic memory in later life (fully adjusted).

Predictors	Uncorrected	Bias-corrected	Error-corrected
Constant	$11.722 \pm 2.118^{\dagger}$	$12.140 \pm 2.118^{\dagger}$	$11.894 \pm 2.118^{\dagger}$
Sex, Female	$0.772 \pm 0.088^{\dagger}$	$0.768 \pm 0.088^{\dagger}$	$0.765 \pm 0.088^{\dagger}$
Age	-0.047 ± 0.058	-0.048 ± 0.058	-0.050 ± 0.058
Age^2	-0.001 ± 0.000	-0.001 ± 0.000	-0.001 ± 0.000
Household size	0.037 ± 0.052	0.036 ± 0.052	0.036 ± 0.052
Routine manual	$-0.632 \pm 0.095^{\dagger}$	$-0.622 \pm 0.095^{\dagger}$	$-0.618 \pm 0.095^{\dagger}$
Wealth mid-tertile	$0.308 \pm 0.109^{\dagger}$	$0.303 \pm 0.109^{\dagger}$	$0.299 \pm 0.109^{\dagger}$
Wealth top-tertile	$0.596 \pm 0.116^{\dagger}$	$0.585 \pm 0.116^{\dagger}$	$0.579 \pm 0.116^{\dagger}$
Age left education	$0.185 \pm 0.019^{\dagger}$	$0.180 \pm 0.019^{\dagger}$	$0.177 \pm 0.019^{\dagger}$
Married-cohab.	0.105 ± 0.203	0.106 ± 0.203	0.105 ± 0.203
Separated-div.	0.137 ± 0.230	0.135 ± 0.229	0.130 ± 0.229
Widow	0.156 ± 0.219	0.156 ± 0.219	0.156 ± 0.219
Social support	0.173 ± 0.399	0.176 ± 0.399	0.175 ± 0.399
Social connection	$0.064 \pm 0.012^{\dagger}$	$0.063 \pm 0.012^{\dagger}$	$0.063 \pm 0.012^{\dagger}$
CVD	-0.083 ± 0.108	-0.084 ± 0.108	-0.085 ± 0.108
COPD	0.170 ± 0.116	0.172 ± 0.116	0.174 ± 0.116
Cancer	0.333 ± 0.177	0.328 ± 0.177	0.323 ± 0.177
Smoking	$-0.351 \pm 0.129^{\dagger}$	$-0.346 \pm 0.129^{\dagger}$	$-0.342 \pm 0.129^{\dagger}$
Drinking	$0.477 \pm 0.091^{\dagger}$	$0.474 \pm 0.091^{\dagger}$	$0.474 \pm 0.091^{\dagger}$
Exercise, vigorous	0.085 ± 0.109	0.086 ± 0.109	0.087 ± 0.109
Exercise, moderate	$0.384 \pm 0.091^{\dagger}$	$0.384 \pm 0.091^{\dagger}$	$0.383 \pm 0.091^{\dagger}$
CESD	$-0.116 \pm 0.024^{\dagger}$	$-0.116 \pm 0.024^{\dagger}$	$-0.116 \pm 0.024^{\dagger}$
Childhood lack facility	-0.054 ± 0.029	$-0.191 \pm 0.029^{\dagger}$	$-0.273 \pm 0.118^{*}$
RMSE	0.0406	0.0092	0.0405

Source: The English Longitudinal Study of Ageing.

* $p < 0.05$.

† $p < 0.01$.

Table C. Three models on mental health in later life (fully adjusted).

Predictors	Uncorrected	Bias-corrected	Error-corrected
Constant	2.185 ± 1.229	$2.450 \pm 1.229^{*}$	2.183 ± 1.230
Sex, Female	$-0.318 \pm 0.051^{\dagger}$	$-0.320 \pm 0.051^{\dagger}$	$-0.317 \pm 0.051^{\dagger}$
Age	0.063 ± 0.034	0.062 ± 0.034	0.063 ± 0.034
Age^2	0.000 ± 0.000	0.000 ± 0.000	0.000 ± 0.000
Household size	$0.060 \pm 0.030^{*}$	0.060 ± 0.030	$0.060 \pm 0.030^{*}$
Routine manual	-0.097 ± 0.055	-0.091 ± 0.055	-0.097 ± 0.055
Wealth mid-tertile	$0.287 \pm 0.063^{\dagger}$	$0.283 \pm 0.063^{\dagger}$	$0.287 \pm 0.063^{\dagger}$
Wealth top-tertile	$0.517 \pm 0.067^{\dagger}$	$0.510 \pm 0.067^{\dagger}$	$0.517 \pm 0.067^{\dagger}$
Age left education	0.009 ± 0.011	0.006 ± 0.011	0.009 ± 0.011
Married-cohab.	0.115 ± 0.118	0.116 ± 0.118	0.115 ± 0.118
Separated-div.	-0.211 ± 0.133	-0.213 ± 0.133	-0.212 ± 0.133
Widow	$-0.502 \pm 0.127^{\dagger}$	$-0.502 \pm 0.127^{\dagger}$	$-0.502 \pm 0.127^{\dagger}$
Social support	$1.107 \pm 0.232^{\dagger}$	$1.109 \pm 0.232^{\dagger}$	$1.108 \pm 0.232^{\dagger}$
Social connection	$0.030 \pm 0.007^{\dagger}$	$0.030 \pm 0.007^{\dagger}$	$0.030 \pm 0.007^{\dagger}$
CVD	-0.104 ± 0.063	-0.105 ± 0.063	-0.104 ± 0.063
COPD	$-0.474 \pm 0.067^{\dagger}$	$-0.473 \pm 0.067^{\dagger}$	$-0.474 \pm 0.067^{\dagger}$
Cancer	-0.115 ± 0.103	-0.118 ± 0.103	-0.115 ± 0.103
Smoking	$-0.255 \pm 0.075^{\dagger}$	$-0.252 \pm 0.075^{\dagger}$	$-0.255 \pm 0.075^{\dagger}$
Drinking	$0.132 \pm 0.053^{*}$	$0.130 \pm 0.053^{*}$	$0.132 \pm 0.053^{*}$
Exercise, vigorous	$0.134 \pm 0.064^{*}$	$0.134 \pm 0.064^{*}$	$0.134 \pm 0.064^{*}$
Exercise, moderate	$0.504 \pm 0.053^{\dagger}$	$0.504 \pm 0.053^{\dagger}$	$0.504 \pm 0.053^{\dagger}$
Childhood lack facility	-0.005 ± 0.017	$-0.091 \pm 0.017^{\dagger}$	-0.003 ± 0.017
$RMSE$	0.0236	0.0206	0.0236

Source: The English Longitudinal Study of Ageing.

* $p < 0.05$.

† $p < 0.01$.

Table D. Three models on gait speed in later life, with adult health (fully adjusted).

Predictors	Uncorrected	Bias-corrected	Error-corrected
Constant	$0.667 \pm 0.262^{*}$	$0.746 \pm 0.262^{\dagger}$	$0.697 \pm 0.262^{\dagger}$
Sex, Female	$-0.049 \pm 0.008^{\dagger}$	$-0.050 \pm 0.008^{\dagger}$	$-0.050 \pm 0.008^{\dagger}$
Age	0.009 ± 0.007	0.009 ± 0.007	0.008 ± 0.007
Age^2	$0.000 \pm 0.000^{\dagger}$	$0.000 \pm 0.000^{\dagger}$	$0.000 \pm 0.000^{\dagger}$
Household size	0.004 ± 0.006	0.004 ± 0.006	0.004 ± 0.006
Routine manual	$-0.039 \pm 0.009^{\dagger}$	$-0.037 \pm 0.009^{\dagger}$	$-0.037 \pm 0.009^{\dagger}$
Wealth mid-tertile	$0.039 \pm 0.010^{\dagger}$	$0.037 \pm 0.010^{\dagger}$	$0.037 \pm 0.010^{\dagger}$
Wealth top-tertile	$0.081 \pm 0.011^{\dagger}$	$0.080 \pm 0.011^{\dagger}$	$0.079 \pm 0.011^{\dagger}$
Age left edu	$0.006 \pm 0.002^{\dagger}$	$0.005 \pm 0.002^{\dagger}$	$0.005 \pm 0.002^{\dagger}$
Married-cohab.	$0.040 \pm 0.020^{*}$	$0.040 \pm 0.020^{*}$	$0.040 \pm 0.020^{*}$
Separated-div.	0.040 ± 0.023	0.040 ± 0.023	0.040 ± 0.023
Widow	0.033 ± 0.021	0.033 ± 0.021	0.033 ± 0.021
Social support	-0.023 ± 0.036	-0.023 ± 0.036	-0.023 ± 0.036
Social connection	$0.005 \pm 0.001^{\dagger}$	$0.005 \pm 0.001^{\dagger}$	$0.005 \pm 0.001^{\dagger}$
CVD	$-0.021 \pm 0.010^{*}$	$-0.021 \pm 0.010^{*}$	$-0.021 \pm 0.010^{*}$
COPD	$-0.028 \pm 0.011^{\dagger}$	$-0.027 \pm 0.011^{*}$	$-0.027 \pm 0.011^{*}$
Cancer	0.004 ± 0.015	0.003 ± 0.015	0.003 ± 0.015
Smoking	$-0.027 \pm 0.013^{*}$	$-0.027 \pm 0.013^{*}$	$-0.026 \pm 0.013^{*}$
Drinking	$0.018 \pm 0.009^{*}$	$0.017 \pm 0.009^{*}$	$0.017 \pm 0.009^{*}$
Exercise, vigorous	$0.041 \pm 0.011^{\dagger}$	$0.041 \pm 0.011^{\dagger}$	$0.041 \pm 0.011^{\dagger}$
Exercise, moderate	$0.101 \pm 0.008^{\dagger}$	$0.101 \pm 0.008^{\dagger}$	$0.101 \pm 0.008^{\dagger}$
Childhood lack facility	-0.005 ± 0.003	$-0.029 \pm 0.003^{\dagger}$	$-0.035 \pm 0.011^{\dagger}$
Adult illness episodes	$-0.032 \pm 0.004^{\dagger}$	$-0.032 \pm 0.004^{\dagger}$	$-0.032 \pm 0.004^{\dagger}$
$RMSE$	0.0038	0.0006	0.0037

Source: The English Longitudinal Study of Ageing 2006.

* $p < 0.05$.

† $p < 0.01$.

Table E. Three models on episodic memory in later life, with adult health (fully adjusted).

Predictors	Uncorrected	Bias-corrected	Error-corrected
Constant	$11.750 \pm 2.120^\dagger$	$12.174 \pm 2.120^\dagger$	$11.927 \pm 2.120^\dagger$
Sex, Female	$0.772 \pm 0.088^\dagger$	$0.769 \pm 0.088^\dagger$	$0.766 \pm 0.088^\dagger$
Age	-0.048 ± 0.058	-0.050 ± 0.058	-0.052 ± 0.058
Age^2	-0.001 ± 0.000	-0.001 ± 0.000	-0.001 ± 0.000
Household size	0.037 ± 0.052	0.037 ± 0.052	0.037 ± 0.052
Routine manual	$-0.633 \pm 0.095^\dagger$	$-0.623 \pm 0.095^\dagger$	$-0.618 \pm 0.095^\dagger$
Wealth mid-tertile	$0.310 \pm 0.109^\dagger$	$0.305 \pm 0.109^\dagger$	$0.301 \pm 0.109^\dagger$
Wealth top-tertile	$0.598 \pm 0.116^\dagger$	$0.587 \pm 0.116^\dagger$	$0.582 \pm 0.116^\dagger$
Age left edu	$0.184 \pm 0.019^\dagger$	$0.18 \pm 0.0190^\dagger$	$0.177 \pm 0.019^\dagger$
Married-cohab.	0.105 ± 0.203	0.106 ± 0.203	0.105 ± 0.203
Separated-div.	0.137 ± 0.229	0.135 ± 0.229	0.130 ± 0.229
Widow	0.158 ± 0.219	0.158 ± 0.219	0.158 ± 0.219
Social support	0.175 ± 0.399	0.177 ± 0.399	0.177 ± 0.399
Social connection	$0.064 \pm 0.012^\dagger$	$0.063 \pm 0.012^\dagger$	$0.063 \pm 0.012^\dagger$
CVD	-0.084 ± 0.108	-0.086 ± 0.108	-0.086 ± 0.108
COPD	0.166 ± 0.117	0.168 ± 0.117	0.169 ± 0.116
Cancer	0.329 ± 0.178	0.323 ± 0.178	0.319 ± 0.178
Smoking	$-0.351 \pm 0.129^\dagger$	$-0.346 \pm 0.129^\dagger$	$-0.341 \pm 0.129^\dagger$
Drinking	$0.478 \pm 0.091^\dagger$	$0.475 \pm 0.091^\dagger$	$0.475 \pm 0.091^\dagger$
Exercise, vigorous	0.087 ± 0.110	0.088 ± 0.110	0.089 ± 0.110
Exercise, moderate	$0.386 \pm 0.091^\dagger$	$0.386 \pm 0.091^\dagger$	$0.385 \pm 0.091^\dagger$
Childhood lack facility	$-0.118 \pm 0.024^\dagger$	$-0.117 \pm 0.024^\dagger$	$-0.117 \pm 0.024^\dagger$
Adult illness episodes	-0.054 ± 0.029	$-0.192 \pm 0.029^\dagger$	$-0.273 \pm 0.118^*$
RMSE	0.0406	0.0189	0.0405

Source: The English Longitudinal Study of Ageing.

* $p < 0.05$.

† $p < 0.01$.

Table F. Three models on mental health in later life, with adult health (fully adjusted)

Predictors	Uncorrected	Bias-corrected	Error-corrected
Constant	1.676 ± 1.209	1.871 ± 1.209	1.664 ± 1.210
Sex, Female	$-0.317 \pm 0.050^{\dagger}$	$-0.318 \pm 0.050^{\dagger}$	$-0.316 \pm 0.050^{\dagger}$
Age	$0.089 \pm 0.033^{\dagger}$	$0.088 \pm 0.033^{\dagger}$	$0.089 \pm 0.033^{\dagger}$
Age^2	$-0.001 \pm 0.000^{*}$	$-0.001 \pm 0.000^{*}$	$-0.001 \pm 0.000^{*}$
Household size	0.042 ± 0.030	0.042 ± 0.030	0.042 ± 0.030
Routine manual	-0.084 ± 0.054	-0.079 ± 0.054	-0.084 ± 0.054
Wealth mid-tertile	$0.236 \pm 0.062^{\dagger}$	$0.233 \pm 0.062^{\dagger}$	$0.236 \pm 0.062^{\dagger}$
Wealth top-tertile	$0.442 \pm 0.066^{\dagger}$	$0.437 \pm 0.066^{\dagger}$	$0.442 \pm 0.066^{\dagger}$
Age left edu	0.010 ± 0.011	0.008 ± 0.011	0.010 ± 0.011
Married-cohab.	0.109 ± 0.116	0.109 ± 0.116	0.108 ± 0.116
Separated-div.	-0.205 ± 0.131	-0.206 ± 0.131	-0.205 ± 0.131
Widow	$-0.528 \pm 0.125^{\dagger}$	$-0.528 \pm 0.125^{\dagger}$	$-0.529 \pm 0.125^{\dagger}$
Social support	$1.037 \pm 0.228^{\dagger}$	$1.038 \pm 0.228^{\dagger}$	$1.037 \pm 0.228^{\dagger}$
Social connection	$0.030 \pm 0.007^{\dagger}$	$0.030 \pm 0.007^{\dagger}$	$0.030 \pm 0.007^{\dagger}$
CVD	-0.071 ± 0.062	-0.072 ± 0.062	-0.071 ± 0.062
COPD	$-0.359 \pm 0.066^{\dagger}$	$-0.358 \pm 0.066^{\dagger}$	$-0.359 \pm 0.066^{\dagger}$
Cancer	-0.005 ± 0.102	-0.008 ± 0.102	-0.005 ± 0.102
Smoking	$-0.262 \pm 0.074^{\dagger}$	$-0.260 \pm 0.074^{\dagger}$	$-0.262 \pm 0.074^{\dagger}$
Drinking	0.099 ± 0.052	0.098 ± 0.052	0.100 ± 0.052
Exercise, vigorous	0.079 ± 0.063	0.080 ± 0.063	0.079 ± 0.063
Exercise, moderate	$0.443 \pm 0.052^{\dagger}$	$0.443 \pm 0.052^{\dagger}$	$0.443 \pm 0.052^{\dagger}$
Childhood lack facility	-0.011 ± 0.016	$-0.074 \pm 0.016^{\dagger}$	-0.008 ± 0.017
Adult illness episodes	$-0.318 \pm 0.024^{\dagger}$	$-0.317 \pm 0.024^{\dagger}$	$-0.318 \pm 0.024^{\dagger}$
$RMSE$	0.0232	0.0107	0.0232

Source: The English Longitudinal Study of Ageing.

* $p < 0.05$.

† $p < 0.01$.
