# Supplementary material for: Growing Up in Poverty, Growing Old in Infirmity: The Long Arm of Childhood Conditions in Great Britain
Source: PLoS One. 2015 Dec 16;10(12):e0144722. doi: 10.1371/journal.pone.0144722 (PMC4682716; doi:10.1371/journal.pone.0144722)
Supplement: S1 Text — (DOCX) [file pone.0144722.s002.docx]

**S1 Text. Attrition and a few other methodological problems in the study of cognitive ageing**

Gindo Tampubolon

**Abstract**

**Background**

Dementias in high income countries are set to be the third major burden of disease even as older people are increasingly required to think for themselves how to provide for their lives in retirement. Meanwhile the period of older age continues to extend with increase in life expectancy. This challenge demands an understanding of how cognition changes over an extended period in later life. But studying cognitive ageing in the population faces a difficulty from the fact that older respondents are liable to leave (attrite) before study completion. This study tested three hypotheses: trajectories of cognitive ageing in Britain show an improvement beyond the age of 50; and they are lifted by secular improvement in cognition across cohorts; lastly they are susceptible to distortion due to attrition.

**Methods and Findings**

Using the English Longitudinal Study of Ageing, this paper studied trajectories of episodic memory of Britons aged 50-89 from 2002 to 2013 ($N=5931$). Using joint models the analysis found that levels of episodic memory follow a curvilinear shape, not a steady decline, in later life. The findings also revealed secular improvement in cognitive ageing such that as a cohort is being replaced episodic memory levels in the population improve. The analysis lastly demonstrated that failure to simultaneously model attrition can produce distorted pictures of cognitive ageing.

**Conclusion**

Old age in this century is not necessarily a period dominated by cognitive decline. In identifying behavioural factors associated with better cognitive ageing, such as social connections of traditional and online kinds, the paper raises possibilities of mustering an adequate response to the cognition challenge.

**Statistical analysis**

Longitudinal cognitive ageing studies face two major empirical issues: practice effect in cognitive testing and attrition. Following many excellent analyses of cognitive ageing in Britain and the US (McArdle et al 2007, Rabbitt et al 2001, 2004) we first used maximum likelihood estimator of growth curve model with a missing at random assumption; this is variously known as random coefficients or mixed models. They are often used since they give consistent estimates if attritions are missing at random. This means that, given the observed history of covariates and dependent variables, those who left differ from those who stayed only in a random fashion (Rabbitt et al 2001, 2004).

Episodic memory changes were modelled using a sequence of three models. In all models, practice effect was estimated by entering an indicator of repeat testing following the literature (Rabbitt et al 2001, 2004, McArdle et al 2007, Karlamangla et al 2009, Staff et al 2014). This remains the first choice for an ongoing longitudinal ageing study such as ELSA.

First, an initial model included age, squared age, sex, social determinants, chronic conditions and behavioural risk factors (*baseline model*); then cohort indicators were included in the *cohort model*.

We argued above that a *joint model* of cognition and attrition is needed (Rabbitt et al 2001, Graham et al 2011, Tampubolon 2015). Following a recent study which analysed this ELSA sample using joint models (Tampubolon 2015), we used joint models where the random effects ($h$(.) below) influence both episodic memory, $y$, and attrition, $t$; given these, episodic memory and attrition are independent. One part of the joint model is made up of the growth curve model ($f$(.)); the other part is a survival model ($g$(.)) with sex, age polynomial of degree three and the random intercepts from part one. The likelihood is (Graham et al 2011, Tampubolon 2015)

$L=\int f(y_{i}|\text{b}_{i},\text{x}_{i})g(t_{i}|\text{b}_{i},\text{x}_{i})h(\text{b}_{i})\text{d}b_{i}.$ (1)

Joint models have been found to give robust estimates of cognitive ageing in Australia (Graham et al 2011). Joint models with similar specification have been successfully applied to this sample in explaining older Britons' quality of life (Tampubolon 2015). The robustness of the *cohort model* to attrition can be readily assessed by comparing its results with that of the *joint model*.

**References**

Petra L. Graham, Louise M. Ryan and Mary A. Luszcz. Joint modelling of survival and cognitive decline in the Australian Longitudinal Study of Ageing. *Journal of the Royal Statistical Society, Series C*, 60(2):221-238, 2011.

Arun S. Karlamangla, Dana Miller-Martinez, Carol S. Aneshensel, Teresa E. Seeman, Richard G. Wight and Joshua Chodosh. Trajectories of cognitive function in late life in the United States: Demographic and socioeconomic predictors. *American Journal of Epidemiology*, 170(3):331-342, 2009.

John J. McArdle, Gwenith G. Fisher and Kelly M. Kadlec. Latent variable analyses of age trends of cognition in the Health and Retirement Study, 1992-2004.  *Psychology and Aging*, 22(3):525-545, 2007.

Patrick Rabbitt, Peter Diggle, Fiona Holland and Lynn McInnes. Practice and drop-out effects during a 17-year longitudinal study of cognitive aging. *The Journals of Gerontology Series B: Psychological Sciences and Social Sciences*, 59(2):P84-P97, 2004.

Patrick Rabbitt, Peter Diggle, David Smith, Fiona Holland and Lynn Mc Innes. Identifying and separating the effects of practice and of cognitive ageing during a large longitudinal study of elderly community residents. *Neuropsychologia*, 39(5):532 - 543, 2001.

Roger T. Staff, Michael J. Hogan and Lawrence J. Whalley. Aging trajectories of fluid intelligence in late life: The influence of age, practice and childhood IQ on Raven's Progressive Matrices. *Intelligence*, 47:194-201, 2014.

Gindo Tampubolon. Delineating the Third age: Joint models of older people’s quality of life and attrition in Britain 2002-2010. *Aging & Mental Health*, 19(7):576-583, 2015.
